# Transcriptomic Profiles of MV4-11 and Kasumi 1 Acute Myeloid Leukemia Cell Lines Modulated by Epigenetic Modifiers Trichostatin A and 5-Azacytidine

**Published:** 2020-01-01

**Authors:** Mat Jusoh Siti Asmaa, Hamid Ali Al-Jamal, Abdul Rahim Hussein, Badrul Hisham Yahaya, Roslin Hassan, Faezahtul Arbaeyah Hussain, Shaharum Shamsuddin, Muhammad Farid Johan

**Affiliations:** 1 Department of Hematology, School of Medical Sciences, Universiti Sains Malaysia, 16150 Kubang Kerian, Kelantan, Malaysia; 2 Diagnostic and Biomedicine, Faculty of Health Sciences, Universiti Sultan Zainal Abidin, Gong Badak Campus, Kuala Nerus, 21300, Terengganu, Malaysia; 3 Regenerative Medicine Cluster, Advanced Medical and Dental Institute, Universiti Sains Malaysia, Bertam, 13200 Kepala Batas, Pulau Pinang, Malaysia; 4 Department of Internal Medicine, School of Medical Sciences, Universiti Sains Malaysia, 16150 Kubang Kerian, Kelantan, Malaysia; 5 Department of Pathology, School of Medical Sciences, Universiti Sains Malaysia, 16150 Kubang Kerian, Kelantan, Malaysia; 6 School of Health Sciences, Universiti Sains Malaysia, 16150 Kubang Kerian, Kelantan, Malaysia; 7 Institute for Research in Molecular Medicine (INFORMM), Universiti Sains Malaysia, 16150 Kubang Kerian, Kelantan, Malaysia

**Keywords:** Acute myeloid leukemia, Epigenetics* Histone deacetylase inhibitors, 5-Azacytidine, Gene expression

## Abstract

**Background:** Acute myeloid leukemia (AML) is the most common form of acute leukemias in adults which is clinically and molecularly heterogeneous. Several risk and genetic factors have been widely investigated to characterize AML. However, the concomitant epigenetic factors in controlling the gene expression lead to AML transformation was not fully understood. This study was aimed to identify epigenetically regulated genes in AML cell lines induced by epigenetic modulating agents, Trichostatin A (TSA) and 5-Azacytidine (5-Aza).

**Materials and Methods:** MV4-11 and Kasumi 1 were treated with TSA and/or 5-Aza at IC_50_ concentration. Gene expression profiling by microarray was utilized using SurePrint G3 Human Gene Expression v3. Gene ontology and KEGG pathway annotations were analyzed by DAVID bioinformatics software using EASE enrichment score. mRNA expression of the differentially expressed genes were verified by quantitative real time PCR.

**Results:** Gene expression analysis revealed a significant changes in the expression of 24,822, 15,720, 15,654 genes in MV4-11 and 12,598, 8828, 18,026 genes in Kasumi 1, in response to TSA, 5-Aza and combination treatments, respectively, compared to non-treated (*p*<0.05). 7 genes (*SOCS3*, *TUBA1C*, *CCNA1*, *MAP3K6*, *PTPRC*, *STAT6* and *RUNX1*) and 4 genes (*ANGPTL4*, *TUBB2A*, *ADAM12* and *PTPN6*) shown to be predominantly expressed in MV4-11 and Kasumi 1, respectively (EASE<0.1). The analysis also revealed phagosome pathway commonly activated in both cell lines.

**Conclusion:** Our data showed a distinct optimal biological characteristic and pathway in different types of leukemic cell lines. These finding may help in the identification of cell-specific epigenetic biomarker in the pathogenesis of AML.

## Introduction

Acute myeloid leukemia (AML) is characterized by a block in early progenitor differentiation leading to accumulation of immature and highly proliferative leukemic stem cells (LSCs) in the bone marrow and peripheral blood^   1 ^. The 2017 World Health Organization (WHO) has provided guidelines on the cut-off value of blast percentage of AML by; 200 and 500 cells-leukocytes differential counts in the peripheral blood and in the bone marrow, respectively^   2 ^. For a diagnosis of AML, a marrow or blood blast count of 20% or more is required, except for AML with t(15;17), t(8;21), inv(16) or t(16;16), and some cases of erythroleukemia. AML is the most common form of acute leukemias in adults which affected 32% adults. Although the overall mortality rate has decreased by 1.0% each year from 2001 to 2010, the overall incidence rate was increased by 0.2% each year. In 2018, the American Cancer Society estimated that 19,520 of new cases and 10,670 deaths from AML. The 5-years overall survival rate was also poor with only 24%^   3 ^. 

For many years, gene expression profiling by microarray was used as a traditional method to search abnormalities in cancers, including in AML^   4 ^. These presented data was invaluable and accessible to the identification of disease’s class discovery, class prediction, and class comparison. Class discovery refers to the identification of a new subgroup, that later was class predicted by gene expression data. The first and second class already had a diagnostic implication. While the third class, which is class comparison refer to the identification of genes that were deregulated in certain subgroups, that may address biological function^   5 ^. 

It has long established that AML is clinically heterogeneous disease characterized by an accumulation of continuous genetic abnormalities^   6 ^ and prior epigenetic lesions^   7 ^ resulting in clonal evolution and expansion. The considerable complexities disrupt the genetic and epigenetic landscapes by changes in gene expression^   8 ^ which profoundly affecting treatment response and patients’ survival. Earlier epigenetic alteration established cellular identities initiating tumorigenesis by inappropriate activation or inhibition of cellular signaling pathways^   9 ^. For example, promoter hypermethylation of a tumor suppressor genes is commonly implicated in cancer^   10 ^, involving genes controlling the cell cycle and DNA repair^   11 ^. On the other hand, modification to histone protein in nucleosome modulates the transcriptional burst frequency specifically through histone acetylation^   12 ^. Both epigenetic mechanisms endow the regulation in gene expression. Hence, targeting the epigenetically-regulated genes in the control of AML licensed a promising outcome. 

In this study, high-throughput microarray technique was used to analyze epigenetic-derived molecular mechanism by modulating gene expression using a classical DNA methyltransferase (DNMT) inhibitor; 5-Azacytidine (5-Aza) and a histone deacetylase (HDAC) inhibitor, Trichostatin A (TSA). The aim of this study was to induce the epigenetic response via gene re-expression or down-expression in two types of AML cell lines; MV4-11 and Kasumi 1. It was hypothesized that the silencing of a tumor suppressor gene and the activation of oncogenes in AML were due to epigenetic mechanisms of DNA hypermethylation and histone deacetylation. 

## MATERIALS AND METHODS

**MV4-11 and Kasumi 1 cell culture**

MV4-11 is a human AML cell line established from blasts cells of 10 years old male with biphenotypic B-myelomonocytic leukemia (AML FAB M5) that carry translocation t(4;11) and a *FLT3*-ITD mutation. Kasumi 1 is a human AML cell line established from peripheral blast cells from 7 years old juvenile male Japanese that carry translocation t(8;21) and *AML1-ETO *(also known as *RUNX1-CBF2T1*) fusion genes. The AML cell lines were originally purchased from the American Type Culture Collection (ATCC, VA, USA). Both AML cell lines were cultured in RPMI-1640 (Gibco^®^, CA, USA) supplemented with 10% Fetal bovine serum (Sigma-Aldrich, MO, USA) and 0.1% penicillin/streptomycin (Invitrogen, CA, USA) in humidified temperature containing 5% carbon dioxide (CO_2_) at 37°C. 

**TSA and/or 5-Aza treatment**

TSA (Sigma-Aldrich, MO, USA) and 5-Aza (Sigma-Aldrich, MO, USA) were dissolved in DMSO (Sigma-Aldrich, MO, USA) and RPMI-1640, respectively to a stock concentration of 500 µM, and further diluted to the desired working concentrations. MV4-11 and Kasumi 1 were seeded in 6-wells plate to 80-90% confluency at the initial cell number of 1 x 10^5^ cells/mL prior to the drug treatment for 24 hours. The cell lines were treated with varying concentration of TSA (0, 1.25, 2.5, 5.0, 10.0 µM) and 5-Aza (0, 5.0, 10.0, 20.0, 50, 100 µM) and incubated for 24 hours under humidified temperature. 

**Cell Viability Assay**

Percentage viability of non-treated and treated MV4-11 and Kasumi 1 after the 24 hours exposure to TSA and 5-Aza treatments were measured by Trypan Blue Exclusion Assay (Life Technologies, CA, USA). The half maximal inhibitory concentration (IC_50_) was determined by GraphPad Prism 6.0 (GraphPad, CA, USA). 

**Total RNA extraction and quality control**

Total RNA was extracted from treated and untreated MV4-11 and Kasumi 1 using Total RNA Isolation Kit (Promega, SA, USA) according to the manufacturer’s protocol. The final elution step was performed using 30 µl of elution buffer for a highly concentrated RNAs. The isolated RNA concentration and purity were determined by Nanodrop ND-1000 spectrophotometer (Thermo-Fisher Scientific, WA, USA). Prior to the gene expression profiling, the RNA integrity was assessed by 1.5% agarose gel electrophoresis and their RIN (RNA integrity number) values were determine by Agilent 2100 Bioanalyzer (Agilent, CA, USA). The qualified RNAs (absorbance 280/260 1.8-2.1 ratio; highly intact 28S and 18S ribosomal RNA and RIN above 7) were stored at -80 ºC until further analysis.

**Microarray analysis**

Whole genome expression profiling was performed using One-Color SurePrint G3 Human Gene Expression v3, 8 x 60K slides contained array probe (Agilent Technologies, CA, USA). Prior to Cyanine 3 (Cy3) labeling, RNA spiked-In dilution was prepared using RNA spiked-In Kit (Agilent Technologies, CA, USA) to each sample using T7 RNA polymerase (RNA reference target) for normalization. Cy3-labeled cRNA was generated from 25 ng input total RNA using Low Input Quick Amp Labeling Kit (Agilent Technologies, CA, USA). The fluorescent-labeled cRNA was purified by RNAeasy Mini Kit and RNAase-free DNAase Set (Qiagen, CA, USA) and quantified by Nanodrop ND-1000 spectrophotometer. 25 ng of fluorescein-labeled and amplified cRNA was hybridized into array slides containing 60,000 probes (Agilent Technologies, CA, USA) at 65 degree Celsius for 17 hours. After hybridization and washing steps, the array slides were scanned using SureCan Microarray Scanner (Agilent Technologies, CA, USA) to measure the fluorescence intensity of Cy3 labeled RNA bound to the microarray slide. The resulted images were processed using the Feature Extraction (FE) software v.12 (Agilent Technologies, CA, USA) for data filtering. Raw data obtained was analyzed by Genespring GX v12.6 software (Agilent Technologies, CA, USA).

**Database screening**

Gene Ontology (GO) and Kyoto Encyclopedia of Genes and Genomes (KEGG) pathway analysis annotations were utilized by the Database for Annotation, Visualization and Integrated Discovery (DAVID) Bioinformatics Resources v6.8 (https://david.ncifcrf.gov/) to characterize and predict epigenetically regulated genes in treated AML cell lines. The Enhanced AL Scoring Engine (EASE) scoring system (a modified Fisher Exact p-value, p<0.1) was implemented for statistical analysis to provide enriched GO terms and pathways annotation within gene lists. EASE analysis produces a consistent and similar functional annotation with numerous analytical methods^   13 ^, and Venn diagram was constructed to analyze genes with differential expression pattern after TSA and 5-Aza treatment in MV4-11 and Kasumi 1. The analysis was conducted by the Venny 2.1 software (http://bioinfogp.cnb.csic.es/tools/venny/). 

**Quantitative Real-time PCR (qRT-PCR)**

To validate microarray data, qRT-PCR analysis on selected up-regulated and down-regulated genes was performed by Taqman gene expression assays and analyzed using Applied Biosystem (ABI)® 7500 Real-Time PCR Machine (Applied Biosystem, CA, USA). Total RNAs from untreated and treated cell lines were reverse transcribed using High-Capacity cDNA Reverse Transcription Kit (Applied Biosystem, CA, USA). Pre-designed assays (PrimeTime® Pre-designed Assays) (IDT Inc., IA, USA) [*ANGPTL4* (assay ID: Hs.PT58.25480012), *TUBB2A* (assay ID: Hs.PT58.40767003), *PTPN6* (assay ID: Hs.PT58.23073507) and *ADAM12* (assay ID: Hs.PT58.26423628)], and custom-designed primers and probes (*SOCS3, TUBA1C, CCNA1, MAP3K6, STAT6, PTPRC* and *RUNX1* genes) were amplified by PrimeTime® Gene Expression Master Mix (IDT Inc., IA, USA). Assay sequences were confirmed using web Basic Local Alignment Search Tool (BLAST) by the National Center for Biotechnology Information (NCBI) (U.S. National Library of Medicine, MD, USA). The qRT-PCR amplification conditions were: 95°C for 3 min for enzyme activation, 40 cycles of denaturation at 95°C for 15 s and 60°C for 1 min for annealing and extension. *B2M* and *GAPDH* were used as endogenous control genes and expression levels were estimated using relative quantitation (RQ) of duplicated samples calculated by 2^-∆∆CT^ method (∆∆CT=∆CT_Treated_–∆CT_Untreated_, ∆CT=Ct_Selected Genes _–Ct_B2M/GAPDH_).

## Results

**Figure 1:**
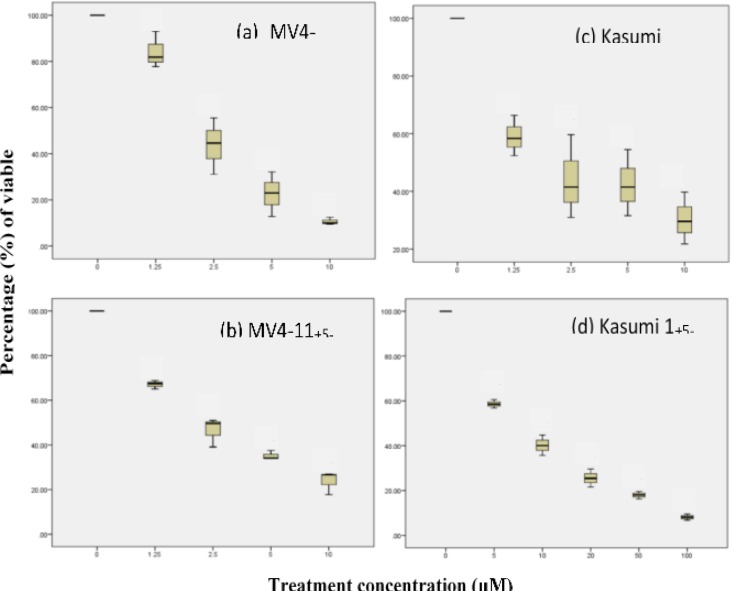
Effect of TSA and 5-Aza treatment on cell viability by percentage (%) inhibition of MV4-11 and Kasumi 1 cell lines relative to non-treated cell lines. Significant inhibition of MV4-11 after (a) TSA and (b) 5-Aza treatment at increasing concentration (0.0, 1.25, 2.5, 5.0 and 10.0 µM) for 24 h. Significant inhibition of Kasumi 1 after (c) TSA treatment at increasing concentration (0.0, 1.25, 2.5, 5.0 and 10.0 µM) and (d) 5-Aza (0.0, 5.0, 10.0, 20.0, 50.0 and 100.0 µM) for 24 h calculated by Trypan Blue Exclusion Assay (TBEA) (One-Way ANOVA, LSD multiple comparison, *p*<0.05).

A significant decrease in cell viability was observed after the TSA and 5-Aza treatments (One-way ANOVA, p<0.05). The half maximal inhibitory concentration (IC_50_) was acquired at 2.2 µM and 2.3 µM for MV4-11 and; 6.25 µM and 6.95 µM for Kasumi 1 in TSA and 5-Aza, respectively. TSA and 5-Aza treatments have higher potency in MV4-11 due to their lower IC_50_ value compared to Kasumi 1 (Figure 1). 

**Gene expression profile of MV4-11 and Kasumi 1 in response to TSA and 5-Aza**

**Figure 2:**
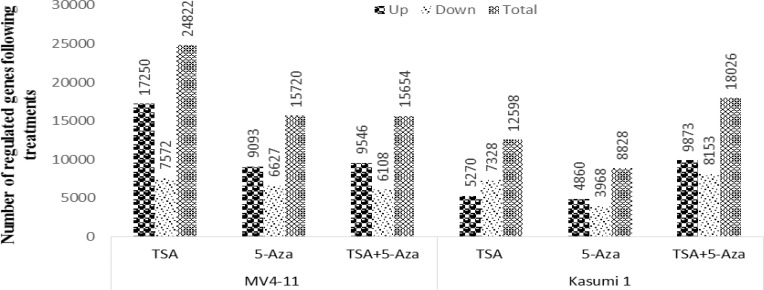
Microarray gene expression analysis for MV4-11 and Kasumi 1 treated with TSA, 5-Aza and TSA+5-Aza. Number of up-regulated and down-regulated genes was created by Genespring software analysis. Further analysis to obtain gene entities were performed using Moderated T-test with multiple correction (Benjamini Hochberg FDR) with *p*-value <0.05 and fold change of >2.0 as a significant.

The gene expression profile of MV4-11 and Kasumi 1 after 24 hours of TSA, 5-Aza and combination (TSA+5-Aza) treatments at IC_50_ concentration. The exploratory microarray analysis was carried out to short-list the differentially expressed genes induced by the drug treatments analyzed by GeneSpring software 12.1 (the cut-off value; fold change ≥ 2.0, significance level, Pearson, P <0.05). 33,150 and 24,668 genes passed the FE filtering in MV4-11 and Kasumi 1, respectively. In MV4-11, 24,822 genes’ expressions were altered (either up or down-regulated) in TSA, 15,720 in 5-Aza and 15,654 in TSA+5-Aza. Whereas in Kasumi 1, 12,598 genes were altered in TSA, 8828 genes in 5-Aza and 18,026 genes in TSA+5-Aza treatments, normalized to non-treated cells (Figure 2). The most up-regulated and down-regulated genes in TSA, 5-Aza and TSA+5-Aza treatments and their folds change were listed in Tables 1 and 2. Genes were selected according to these three criteria: 1. Relevant genes with the highest fold-change different and commonly regulated across all treatments, 2. Relevant genes reported having an association with AML and other myeloid neoplasms from the previous study and/or Pubmed literature, 3. Genes with not otherwise classified under both criteria but could be interesting due to their implication in pathways in cancer.

**Table 1(a):** Most up- and down-regulated genes in TSA treated MV4-11

**Gene Bank ** **Accession**	**Gene symbol**	**Gene description (** **Homo sapiens)**	*Folds **Change**
NM_001082	*CYP4F2*	Cytochrome P450, family 4, subfamily F, polypeptide 2	1094.05
NM_014971	*EFR3B*	EFR3 homolog B (S. cerevisiae)	360.59
NM_006569	*CGREF1*	Cell growth regulator with EF-hand domain 1	348.85
NM_017702	*DEF8*	Differentially expressed in FDCP 8	325.92
NM_003914	*CCNA1*	Cyclin A1	298.44
NM_003255	*TIMP2*	TIMP metallopeptidase inhibitor 2	281.56
NM_031313	*ALPPL2*	Alkaline phosphatase, placental-like 2	250.36
NM_032704	*TUBA1C*	Tubulin, alpha 1c	234.14
NM_003955	*SOCS3*	Suppressor of cytokine signaling 3	176.76
NM_001204054	NDUFC2	NADH dehydrogenase (ubiquinone) 1, subcomplex unknown 2	166.94
NR_027028	*GUSBP1*	Glucuronidase, beta pseudogene 1	153.18
NM_004522	*KIF5C*	Kinesin family member 5C	153.59
NM_003520	*HIST1H2BN*	Histone cluster 1, H2bn	150.13
NM_006321	*ARIH2*	Ariadne RBR E3 ubiquitin protein ligase 2	133.61
NM_000612	*IGF2*	Insulin-like growth factor 2	131.09
NM_177424	*STX12*	Syntaxin 12	103.73
NM_006086	*TUBB3*	Tubulin, beta 3 class III	80.38
NM_004672	*MAP3K6*	Mitogen-activated protein kinase kinase kinase 6	39.50
NM_001025300	RAB12	member RAS oncogene family	38.83
NM_139314	*ANGPTL4*	Angiopoietin-like 4	26.79
NM_018437	*HEMGN*	Hemogen	-518.75
NM_024913	*CPED1*	Cadherin-like and PC-esterase domain containing 1	-243.96
NM_003152	*STAT5A*	Signal transducer and activator of transcription 5A	-159.83
NM_002838	*PTPRC*	Protein tyrosine phosphatase, receptor type C	-138.75
NM_080612	*GAB3*	GRB2-associated binding protein 3	-117.26
NM_003126	*SPTA1*	Spectrin, alpha, erythrocytic 1	-107.30
NM_015401	*HDAC7*	Histone deacetylase 7	-88.16
NM_006563	*KLF1*	Kruppel-like factor 1 (erythroid)	-85.08
NM_015660	*GIMAP2*	GTPase, IMAP family member 2	-73.83
NM_006163	*NFE2*	Nuclear factor, erythroid 2	-69.24
NM_213674	*TPM2*	Tropomyosin 2 (beta)	-57.76
NM_006287	*TFPI*	Tissue factor pathway inhibitor	-55.30
NM_005021	*ENPP3*	pyrophosphatase/phosphodiesterase 3	-49.49
NM_004688	*NMI*	N-myc (and STAT) interactor	-47.85
NM_000037	*ANK1*	Ankyrin 1, erythrocytic, transcript variant 3	-46.78
NM_013427	*ARHGAP6*	Rho GTPase activating protein 6	-42.54
NM_006546	*IGF2BP1*	Insulin-like growth factor 2 mRNA binding protein 1	-42.54
NM_033306	*CASP4*	Caspase 4, apoptosis-related cysteine peptidase	-42.42
NM_080588	*PTPN7*	Protein tyrosine phosphatase, non-receptor type 7	-39.69
NM_004753	*DHRS3*	dehydrogenase/reductase (SDR family) member 3	-36.59
NR_026812	RUNX1-IT1	RUNX1 intronic transcript 1	-22.05
NM_003153	*STAT6*	signal transducer and activator of transcription 6	-10.04

*Folds-change of treatment group compared to control analyzed by Genespring software analysis, Moderated T-test, p<0.05)

**Table 1(b):** Most up- and down-regulated genes in 5-Aza treated MV4-11

**Gene Bank Accession**	**Gene symbol**	**Gene description (** **Homo sapiens)**	*Folds **change**
NM_001145191	*FAM200B*	family with sequence similarity 200, member B	461.79
NM_032905	*RBM17*	RNA binding motif protein 17	336.98
NM_017702	*DEF8*	differentially expressed in FDCP 8 homolog	277.69
NM_024097	*C1orf50*	chromosome 1 open reading frame 50	207.14
NM_001204054	*NDUFC2*	NADH dehydrogenase	185.92
NM_006321	*ARIH2*	ariadne RBR E3 ubiquitin protein ligase 2	158.81
NR_027028	*GUSBP1*	glucuronidase, beta pseudogene 1, non-coding RNA	157.88
NM_032704	*TUBA1C*	tubulin, alpha 1c	154.28
NM_031925	*TMEM120A*	transmembrane protein 120A	135.01
NM_003955	*SOCS3*	suppressor of cytokine signaling 3	120.31
NM_015046	*SETX*	Homo sapiens senataxin	95.04
NM_016256]	*NAGPA*	N-acetylglucosamine-1-phosphodiester alpha-N-acetylglucosaminidase	93.98
NM_001031713	*MCUR1*	mitochondrial calcium uniporter regulator 1	92.49
NM_033028	*BBS4*	Bardet-Biedl syndrome 4	90.09
NM_177424	*STX12*	syntaxin 12	89.59
NM_003520	*HIST1H2BN*	histone cluster 1, H2bn	89.53
NM_052936]	*ATG4A*	autophagy related 4A, cysteine peptidase	85.61
NM_014884	*SUGP2*	SURP and G patch domain containing 2	70.67
NM_138501	*TECR*	trans-2,3-enoyl-CoA reductase	69.28
NM_004672	*MAP3K6*	mitogen-activated protein kinase kinase kinase 6	48.45
NM_005614	*RHEB*	Homo sapiens Ras homolog enriched in brain	45.97
NM_013230	*CD24*	CD24 molecule	45.50
NM_001025300	*RAB12*	RAB12, member RAS oncogene family	44.06
NM_173698	*FAM133A*	family with sequence similarity 133, member A	-101.93
NM_014653	*WSCD2*	WSC domain containing 2	-30.48
NM_145290	*GPR125*	G protein-coupled receptor 125	-29.51
NM_020353	*PLSCR4*	phospholipid scramblase 4	-28.02
NM_001099921	*MAGEB16*	melanoma antigen family B, 16	-27.19
NM_033306	*CASP4*	caspase 4, apoptosis-related cysteine peptidase	-23.01
NM_004126	*GNG11*	guanine nucleotide binding protein (G protein), gamma 11	-22.73
NM_144722	*SPEF2*	sperm flagellar 2	-20.86
NM_015660	*GIMAP2*	GTPase, IMAP family member 2	-19.99
NR_027755	*LINC00922*	long intergenic non-protein coding RNA 922, long non-coding RNA	-19.17
NM_018437	*HEMGN*	hemogen	-18.55
NM_001005285	*OR2AT4*	olfactory receptor, family 2, subfamily AT, member 4	-18.19
NM_000537	*REN*	renin	-17.26
NM_000519	*HBD*	hemoglobin, delta	-16.75
NM_213674	*TPM2*	tropomyosin 2 (beta)	-16.59
NM_002421	*MMP1*	matrix metallopeptidase 1	-12.23
NM_000361	*THBD*	thrombomodulin	-11.98
NM_005807	*PRG4*	proteoglycan 4	-11.81
NM_080429	*AQP10*	aquaporin 10	-11.33
NM_139022	*TSPAN32*	tetraspanin 32	-10.78
NM_024711	*GIMAP6*	GTPase, IMAP family member 6	-10.55
NM_002145	*HOXB2*	homeobox B2	-10.22
NM_019032	*ADAMTSL4*	ADAMTS-like 4	-9.71
NM_002838	*PTPRC*	Protein tyrosine phosphatase, receptor type C	-7.81
NR_026812	RUNX1-IT1	RUNX1 intronic transcript 1	-5.91
NM_003153	*STAT6*	signal transducer and activator of transcription 6	-4.07

**Table 1(c):** Most up- and down-regulated genes in TSA+5-Aza treated MV4-11

**Gene Bank Accession**	**Gene symbol**	**Gene description (** **Homo sapiens)**	*Folds **change**
NM_001145191	FAM200B	Family with sequence similarity 200, member B	521.92
NM_197958	LARP6	La ribonucleoprotein domain family, member 6	506.68
NM_017702	DEF8	differentially expressed in FDCP 8 homolog	268.16
NR_027028	GUSBP1	Homo sapiens glucuronidase, beta pseudogene 1	243.94
NM_032905	RBM17	RNA binding motif protein 17	160.05
NM_014773	KIAA0141	KIAA0141 (KIAA0141)	157.47
NM_001204054	NDUFC2	NADH dehydrogenase (ubiquinone) 1, subcomplex unknown 2	155.54
NM_016256	NAGPA	N-acetylglucosamine-1-phosphodiester alpha-N-acetylglucosaminidase	141.82
NM_032704	TUBA1C	tubulin, alpha 1c	139.42
NM_013268	LGALS13	lectin, galactoside-binding, soluble 13	132.17
NM_004187	KDM5C	lysine (K)-specific demethylase 5C	116.85
NM_024097	C1orf50	chromosome 1 open reading frame 50	113.21
NM_006321	ARIH2	ariadne RBR E3 ubiquitin protein ligase 2	97.43
NM_014035	SNX24	sorting nexin 24	94.35
NM_000600	IL6	interleukin 6 (interferon, beta 2)	91.55
NM_138433	KLHDC7B	kelch domain containing 7B	89.54
NM_033028	BBS4	Bardet-Biedl syndrome 4	87.94
NM_177424	STX12	syntaxin 12	87.27
NM_015046	SETX	senataxin	87.24
NM_001031713	MCUR1	mitochondrial calcium uniporter regulator 1	85.70
NM_001010893	SLC10A5	solute carrier family 10, member 5	79.58
NM_031925	TMEM120A	transmembrane protein 120A	78.16
NM_006945	SPRR2D	small proline-rich protein 2D	71.36
NM_052936	ATG4A	Homo sapiens autophagy related 4A, cysteine peptidase	70.34
NM_014945	ABLIM3	actin binding LIM protein family, member 3	68.78
NM_015701	ERLEC1	endoplasmic reticulum lectin 1	61.29
NM_004672	*MAP3K6*	mitogen-activated protein kinase kinase kinase 6	59.79
NM_006415	SPTLC1	serine palmitoyltransferase, long chain base subunit 1	59.76
NM_001025300	RAB12	RAB12, member RAS oncogene family	59.16
NM_005988	SPRR2A	small proline-rich protein 2A	58.97
NM_001080541	MGA	Homo sapiens MGA, MAX dimerization protein	56.75
NM_144569	SPOCD1	Homo sapiens SPOC domain containing 1	54.22
NM_018357	LARP6	Homo sapiens La ribonucleoprotein domain family, member 6	54.17
NM_206818	OSCAR	osteoclast associated, immunoglobulin-like receptor	53.30
NM_017956	TRMT12	tRNA methyltransferase 12 homolog (S. cerevisiae)	52.10
NM_005614	RHEB	Ras homolog enriched in brain	50.16
NM_012337	CCDC19	coiled-coil domain containing 19	50.03
NM_014884	SUGP2	SURP and G patch domain containing 2	47.37
NM_015335	MED13L	mediator complex subunit 13-like	47.11
NM_173698	FAM133A	family with sequence similarity 133, member A	-153.62
NM_145290	GPR125	G protein-coupled receptor 125	-78.33
NM_017521	FEV	Homo sapiens FEV	-77.72
NM_001541	HSPB2	Homo sapiens heat shock 27kDa protein 2	-67.21
NM_032501	ACSS1	Homo sapiens acyl-CoA synthetase short-chain family member 1	-63.80
NM_021992	TMSB15A	thymosin beta 15a	-55.18
NM_012449	STEAP1	six transmembrane epithelial antigen of the prostate 1	-44.93
NM_017414	USP18	ubiquitin specific peptidase 18	-44.70
NM_001803	CD52	CD52 molecule	-44.63
NM_004126	GNG11	guanine nucleotide binding protein (G protein), gamma 11	-42.81
NM_000519	HBD	hemoglobin, delta	-40.08
NM_033258	GNG8	guanine nucleotide binding protein (G protein), gamma 8	-38.65
NM_138444	KCTD12	potassium channel tetramerization domain containing 12	-35.88
NM_002866	RAB3A	RAB3A, member RAS oncogene family	-35.15
NM_014697	NOS1AP	nitric oxide synthase 1 (neuronal) adaptor protein	-35.11
NM_018437	HEMGN	hemogen	-34.39
NM_207459]	TEX19	testis expressed 19	-33.52
NM_004982	KCNJ8	potassium inwardly-rectifying channel, subfamily J, member 8	-33.13
NM_013251	TAC3	tachykinin 3 222335545766788WWSSF BBGTT	-30.44
NM_032333	FAM213A	family with sequence similarity 213, member A	-29.38
NM_213599	ANO5	anoctamin 5	-29.37
NM_130776	XAGE3	X antigen family, member 3	-28.64
NM_002585	PBX1	pre-B-cell leukemia homeobox 1	-28.42
NM_001110199	SRRM3	Homo sapiens serine/arginine repetitive matrix 3	-28.20
NM_000537	REN	renin	-27.47

*Folds-change of treatment group compared to control analyzed by Genespring software analysis, Moderated T-test, p<0.05)

**Table 2(a):** Most up- and down-regulated genes in TSA treated Kasumi 1

**Gene Bank Accession**	**Gene symbol**	**Gene description ** **(** ** Homo sapiens)**	*Folds **change**
NM_139314	ANGPTL4	angiopoietin-like 4	791.26
NM_182908	DHRS2	dehydrogenase/reductase (SDR family) member 2	612.16
NM_001069	TUBB2A	tubulin, beta 2A class IIa	574.87
NM_001080434	LMTK3	lemur tyrosine kinase 3	356.19
NM_138345	VWA5B2	von Willebrand factor A domain containing 5B2	331.00
NM_030630	HID1	HID1 domain containing	331.00
NM_006928	PMEL	premelanosome protein	323.68
NM_145056	DACT3	dishevelled-binding antagonist of beta-catenin 3	269.03
NM_144698	ANKRD35	ankyrin repeat domain 35,	258.42
NM_014971	EFR3B	EFR3 homolog B (S. cerevisiae)	248.79
NM_004933	CDH15	cadherin 15, type 1, M-cadherin (myotubule)	221.35
NM_006086	TUBB3	tubulin, beta 3 class III	205.73
NM_000088	COL1A1	collagen, type I, alpha 1	122.33
NM_017577	GRAMD1C	GRAM domain containing 1C	109.67
NM_080860	RSPH1	radial spoke head 1 homolog	109.55
NM_003835	RGS9	regulator of G-protein signaling 9	103.85
NM_001098722	GNG4	guanine nucleotide binding protein (G protein), gamma 4	102.41
NM_005325	HIST1H1A	histone cluster 1, H1a	99.67
NM_018667	SMPD3	sphingomyelin phosphodiesterase 3, neutral membrane (neutral sphingomyelinase II)	98.71
NM_033103	RHPN2	rhophilin, Rho GTPase binding protein 2	91.75
NM_007224	NXPH4	neurexophilin 4	88.57
NM_014226	MOK	MOK protein kinase	73.56
NM_001077621	VPS37D	vacuolar protein sorting 37 homolog D	69.03
NM_001145028	PALM3	paralemmin 3	66.97
NM_177403	RAB7B	RAB7B, member RAS oncogene family	-264.07
NM_005574	LMO2	Homo sapiens LIM domain only 2 (rhombotin-like 1)	-215.33
NM_001004196	CD200	CD200 molecule	-162.39
NM_001146	ANGPT1	angiopoietin 1	-159.45
NM_003474	ADAM12	ADAM metallopeptidase domain 12	-137.13
NM_003942	RPS6KA4	Homo sapiens ribosomal protein S6 kinase, 90kDa, polypeptide 4	-136.39
NM_080588	PTPN7	protein tyrosine phosphatase, non-receptor type 7	-133.96
NM_130782	RGS18	regulator of G-protein signaling 18	-119.12
NM_033101	LGALS12	lectin, galactoside-binding, soluble, 12	-94.20
NM_002005	FES	FES proto-oncogene, tyrosine kinase	-93.71
NM_080387	CLEC4D	C-type lectin domain family 4, member D	-93.00
NM_024888	LPPR3	lipid phosphate phosphatase-related protein type 3	-80.70
NM_012252	TFEC	transcription factor EC	-77.90
NM_001805	CEBPE	CCAAT/enhancer binding protein (C/EBP), epsilon	-69.46
NM_014682	ST18	suppression of tumorigenicity 18, zinc finger	-67.63
NM_002467	MYC	v-myc avian myelocytomatosis viral oncogene homolog	-65.46
NM_005263	GFI1	growth factor independent 1 transcription repressor	-64.45
NM_153615	RGL4	ral guanine nucleotide dissociation stimulator-like 4	-63.06
NM_002287	LAIR1	leukocyte-associated immunoglobulin-like receptor 1	-59.78
NM_002586	PBX2	pre-B-cell leukemia homeobox 2	-58.11
NM_005211	CSF1R	colony stimulating factor 1 receptor	-55.40
NM_002831	PTPN6	protein tyrosine phosphatase, non-receptor type 6	-52.38
NM_000442	PECAM1	platelet/endothelial cell adhesion molecule 1	-52.24

*Folds-change of treatment group compared to control analyzed by Genespring software analysis, Moderated T-test, p<0.05)

**Table 2(b):** Most up- and down-regulated genes in 5-Aza treated Kasumi 1

**Gene Bank Accession**	**Gene symbol**	**Gene description ** **(** ** Homo sapiens)**	*Folds **change**
NM_021120	DLG3	discs, large homolog 3 (Drosophila)	14.12
NM_033114	ZCRB1	zinc finger CCHC-type and RNA binding motif 1	12.82
NM_001110514	EBF4	early B-cell factor 4	12.63
NM_013271	PCSK1N	proprotein convertase subtilisin/kexin type 1 inhibitor	11.11
NM_003278	CLEC3B	C-type lectin domain family 3, member B	9.44
NM_003456	ZNF205	zinc finger protein 205	9.23
NM_005252	FOS	FBJ murine osteosarcoma viral oncogene homolog	8.83
NM_002840	PTPRF	protein tyrosine phosphatase, receptor type F	8.83
NM_019058	DDIT4	DNA-damage-inducible transcript 4	8.17
NM_002728	PRG2	proteoglycan 2, bone marrow	7.82
NM_001122962	SIRPB2	signal-regulatory protein beta 2	7.78
NM_001039580	MAP9	microtubule-associated protein 9	7.46
NM_080863	ASB16	ankyrin repeat and SOCS box containing 16	7.21
NM_021158	TRIB3	tribbles pseudokinase 3	6.95
NM_153334	SCARF2	scavenger receptor class F member 2	6.80
NM_002390	ADAM11	ADAM metallopeptidase domain 11	5.63
NM_032797	AIFM2	apoptosis-inducing factor, mitochondrion-associated 2	4.98
NM_004626	WNT11	wingless-type MMTV integration site family, member 11	4.90
NM_032271	TRAF7	TNF receptor-associated factor 7, E3 ubiquitin protein ligase	3.67
NM_001015053	HDAC5	histone deacetylase 5	3.67
NM_001069	TUBB2A	tubulin, beta 2A class IIa	2.67
NM_139314	ANGPTL4	angiopoietin-like 4	2.67
NM_002831	PTPN6	protein tyrosine phosphatase, non-receptor type 6	2.27
NM_001292030	TTC39C	tetratricopeptide repeat domain 39C	-70.59
NM_002844	PTPRK	protein tyrosine phosphatase, receptor type K	-32.81
NM_198481	VSTM1	V-set and transmembrane domain containing 1	-32.49
NM_000099	CST3	cystatin C	-26.47
NM_001244008	KIF1A	kinesin family member 1A	-22.49
NM_001190467	PRR36	proline rich 36	-21.97
NM_024422	DSC2	desmocollin 2	-20.96
NM_001282735	SPATS2L	spermatogenesis associated, serine-rich 2-like	-18.59
NM_015238	WWC1	WW and C2 domain containing 1	-16.52
NM_021199	SQRDL	sulfide quinone reductase-like (yeast)	-15.53
NM_001838	CCR7	chemokine (C-C motif) receptor 7	-13.97
NM_000474	TWIST1	twist family bHLH transcription factor 1	-13.27
NM_012395	CDK14	cyclin-dependent kinase 14	-13.19
NM_000168	GLI3	GLI family zinc finger 3	-12.65
NM_024940	DOCK5	dedicator of cytokinesis 5	-11.91
NM_030906	STK33	serine/threonine kinase 33	-11.90
NM_001900	CST5	cystatin D	-11.86
NM_006897	HOXC9	homeobox C9	-11.74
NM_005855	RAMP1	receptor (G protein-coupled) activity modifying protein 1	-11.55
NM_033292	CASP1	caspase 1, apoptosis-related cysteine peptidase	-11.50
AK027605	CYP2S1	cytochrome P450, family 2, subfamily S, polypeptide 1	-11.02
NM_003474	ADAM12	ADAM metallopeptidase domain 12	-7.681
NM_172217	IL16	interleukin 16	-4.46
NM_001025300	RAB12	RAB12, member RAS oncogene f	-4.89

*Folds-change of treatment group compared to control analyzed by Genespring software analysis, Moderated T-test, p<0.05)

**Table 2(c):** Most up- and down-regulated genes in TSA+5-Aza treated Kasumi 1

**Gene Bank Accession**	**Gene symbol**	**Gene description (** **Homo sapiens)**	*Folds change
NM_182908	DHRS2	dehydrogenase/reductase (SDR family) member 2	758.66
NM_001080434	LMTK3	lemur tyrosine kinase 3	541.34
NM_001069	TUBB2A	tubulin, beta 2A class IIa	435.79
NM_139314	ANGPTL4	angiopoietin-like 4	429.60
NM_138345	VWA5B2	von Willebrand factor A domain containing 5B2	398.46
NM_030630	HID1	HID1 domain containing	341.01
NM_006928	PMEL	premelanosome protein	282.05
NM_014971	EFR3B	EFR3 homolog B (S. cerevisiae)	263.45
NM_144698	ANKRD35	ankyrin repeat domain 35	220.61
NM_145056	DACT3	dishevelled-binding antagonist of beta-catenin	219.77
NM_004933	CDH15	cadherin 15, type 1, M-cadherin	190.60
NM_006086	TUBB3	tubulin, beta 3 class III	173.87
NM_001098722	GNG4	guanine nucleotide binding protein (G protein), gamma 4	167.50
NM_080860	RSPH1	radial spoke head 1 homolog (Chlamydomonas)	146.52
NM_003835	RGS9	regulator of G-protein signaling 9	126.58
NM_007224	NXPH4	neurexophilin 4	124.19
NM_020770	CGN	cingulin	118.29
NM_001145028	PALM3	paralemmin 3	114.39
NM_000088	COL1A1	collagen, type I, alpha 1	111.63
NM_003933	BAIAP3	BAI1-associated protein 3	107.26
NM_017577	GRAMD1C	GRAM domain containing 1C	95.72
NM_052899	GPRIN1	G protein regulated inducer of neurite outgrowth 1	95.72
NM_005325	HIST1H1A	histone cluster 1, H1a	95.08
NM_033141	MAP3K9	mitogen-activated protein kinase kinase kinase 9	92.48
NM_198573	ENHO	energy homeostasis associated	92.06
NM_001039570	KREMEN1	kringle containing transmembrane protein 1	91.54
NM_018667	SMPD3	sphingomyelin phosphodiesterase 3	91.24
NM_012253	TKTL1	transketolase-like 1	87.98
NM_002599	PDE2A	phosphodiesterase 2A, cGMP-stimulated	84.11
NM_033259	CAMK2N2	calcium/calmodulin-dependent protein kinase II inhibitor 2	80.49
NM_014226	MOK	MOK protein kinase	79.66
NM_001678	ATP1B2	ATPase, Na+/K+ transporting, beta 2 polypeptide	78.33
NM_006500	MCAM	melanoma cell adhesion molecule	75.94
NM_001077621	VPS37D	vacuolar protein sorting 37 homolog D	74.87
NM_052924	RHPN1	rhophilin, Rho GTPase binding protein 1	74.59
NM_020127	TUFT1	tuftelin 1	73.36
NM_001040709	SYPL2	synaptophysin-like 2	70.97
NM_032432	ABLIM2	actin binding LIM protein family, member 2	70.76
NM_001024401	SBK1	SH3 domain binding kinase 1	68.42
NM_022742	CCDC136	coiled-coil domain containing 136	68.41
NM_021979	HSPA2	heat shock 70kDa protein 2	67.51
NM_000142	FGFR3	fibroblast growth factor receptor 3	65.65
NM_033103	RHPN2	rhophilin, Rho GTPase binding protein 2	65.01
NM_198196	CD96	CD96 molecule (CD96)	-228.86
NM_001972	ELANE	elastase, neutrophil expressed	-172.59
NM_001244008	KIF1A	kinesin family member 1A	-171.82
NM_133374	ZNF618	zinc finger protein 618	-169.32
NM_020125	SLAMF8	SLAM family member 8	-158.07
NM_003974	DOK2	docking protein 2	-153.14
NM_080387	CLEC4D	C-type lectin domain family 4, member D	-143.62
NM_130782	RGS18	regulator of G-protein signaling 18	-110.02
NM_033101	LGALS12	lectin, galactoside-binding, soluble, 12	-107.48
NM_178443	FERMT3	fermitin family member 3	-106.90
NM_012072	CD93	CD93 molecule	-102.56
NM_001946	DUSP6	dual specificity phosphatase 6	-98.76
NM_012252	TFEC	transcription factor EC	-92.29
NM_002467	MYC	v-myc avian myelocytomatosis viral oncogene homolog	-91.05
NM_001004196	CD200	CD200 molecule	-87.76
NM_005814	GPA33	glycoprotein A33 (transmembrane)	-82.88
NM_153615	RGL4	ral guanine nucleotide dissociation stimulator-like 4	-81.77
NM_080588]	PTPN7	protein tyrosine phosphatase, non-receptor type 7	-79.77
NM_014795	ZEB2	zinc finger E-box binding homeobox 2	-79.47
NM_005211	CSF1R	colony stimulating factor 1 receptor	-74.06
NM_001146	ANGPT1	angiopoietin 1	-70.80
NM_006418	OLFM4	olfactomedin 4	-70.64
NM_014682	ST18	Homo sapiens suppression of tumorigenicity 18	-68.89
NM_177403	RAB7B	RAB7B, member RAS oncogene family	-67.90
NM_198481	VSTM1	V-set and transmembrane domain containing 1	-66.89
NM_005187	CBFA2T3	core-binding factor, runt domain, alpha subunit 2; translocated to, 3	-61.51
NM_003474	ADAM12	ADAM metallopeptidase domain 12	-59.66
NM_005574	LMO2	LIM domain only 2	-58.27
NM_080387	CLEC4D	C-type lectin domain family 4, member D	-54.65
NM_001805	CEBPE	CCAAT/enhancer binding protein (C/EBP), epsilon	-48.73

*Folds-change of treatment group compared to control analyzed by Genespring software analysis, Moderated T-test, p<0.05)

**Identification of an optimal Gene Ontology (GO) and KEGG pathway by DAVID software**

GO analysis identified 13 optimal GO terms in MV4-11 after TSA, 5-Aza and TSA+5-Aza treatments constituted of 7 highly enriched biological processes (BP); Actin filament organization, Cytoskeleton organization, JAK-STAT, Blood coagulation, Positive regulation of activated T cell proliferation, Positive regulation of MAPK cascade and Cytoskeleton-dependent intracellular transport, related to 6 enriched molecular function (MF); GTPase activity, GTP binding, Structural constituent of cytoskeleton, Signal transducer activity, Polysaccharide binding, and Insulin-like growth factor receptor binding. The transduced GO terms were correspondent to 4 enriched KEGG pathway, which was Viral carcinogenesis, Hepatitis B, JAK-STAT and Phagosome (Table 3a). 

**Table 3(a):** Gene ontology (GO) profile after TSA, 5-Aza and TSA+5-Aza treatments in MV4-11

**GO IDs**	**GO term**	**Genes **	***p-value***
	**Biological processes**		
GO:0007015	*Actin filamen organization*	*ARHGAP6, SPTA1, TPM2, TMSB15A*	*0.0084*
*GO:0007010*	*Cytoskeleton organization*	*ABLIM3, TUBA1C, ANK1, TSPAN32, TUBB3*	*0.014*
*GO:0007259*	*JAK-STAT cascade*	*NMI, STAT5A, SOCS3*	*0.015*
*GO:0007596*	*Blood coagulation*	*CYP4F2, HBD, NFE2, THBD, TFPI*	*0.022*
*GO:0042102*	*Positive regulation of activated T cell proliferation*	*CD24, IGF2, IL6*	*0.047*
*GO:0043410*	*positive regulation of MAPK cascade*	*TIMP2, IGF2, IL6*	*0.080*
*GO:0030705*	*Cytoskeleton-dependent intracellular transport*	*KIF5C, TUBA1C*	*0.099*
	**Molecular Functions**		
*GO:0003924*	*GTPase activity*	*GNG11, GNG8, RHEB, RAB3A, TUBA1C, TUBB3*	*0.010*
*GO:0005525*	*GTP binding*	*GIMAP2, GIMAP6, RAB12, RAB3A, RHEB, TUBA1C, TUBB3*	*0.021*
*GO:0005200*	*Structural constituent of cytoskeleton*	*ANK1, SPTA1, TUBA1C, TUBB3*	*0.024*
*GO:0004871*	*Signal transducer activity*	*CD24, GNG11, GNG8, STAT5A, STAT6*	*0.028*
*GO:0030247*	*Polysaccharide binding*	*ENPP3, PRG4*	*0.076*
*GO:0005159*	*Insulin-like growth factor receptor binding*	*IGF2, REN *	*0.081*
	**Pathways**		
	*Viral carcinogenesis*	*CCNA1, HDAC7, HIST1H2BN, STAT5A*	*0.069*
	*Hepatitis B*	*CCNA1, IL6, STAT5A, STAT6*	*0.084*
	*JAK-STAT*	*SOCS3, IL6, STAT5A, STAT6*	*0.084*
	*Phagosome*	*STX12, TUBA1C, TUBB3*	*0.10*

(DAVID software analysis, EASE score 0.1, Benjamini p<0.1)

In Kasumi 1, 16 optimal GO terms by BP were identified; Cell adhesion, Leukocyte migration, Bone mineralization, Regulation of G-protein coupled receptor protein signaling pathway, Positive regulation of cell motility, phagocytosis, Peptidyl-tyrosine dephosphorylation, Protein localization to cell surface, Negative regulation of apoptotic process, Protein phosphorylation, Negative regulation of cell death, Hematopoiesis, Negative regulation of cell proliferation, Response to drug, Angiogenesis and Microtubule-based process, related to 8 MF; Protein tyrosine phosphatase activity, Transmembrane receptor protein tyrosine phosphatase activity, Carbohydrate-binding, Protein kinase activity, Heparin-binding, Protein serine/threonine kinase activity, Beta-catenin binding and Transcription factor binding. The most optimal KEGG pathway induced in Kasumi 1 were; Transcriptional misregulation in cancer, MAPK signaling pathway, PI3K-Akt signaling pathway, Pathways in cancer, Hippo signaling pathway, Proteoglycans in cancer, Ras signaling and Phagosome (Table 3b). 

**Table 3(b):** Gene ontology (GO) profile after TSA, 5-Aza and TSA+5-Aza treatments in Kasumi 1

**GO IDs**	**GO term**	**Genes **	**P-value **
	**Biological processes**		
GO:0007155	*Cell adhesion *	*ADAM12, CDH15, COL1A1, PTPRK, PTPRF, DSC2, ATP1B2, CD96, DSC2, COL1A1, MCAM*	*0.00093*
GO:0050900	*Leukocyte migration*	*ANGPTL1, COL1A1, ATP1B2, PECAM1, PTPN6, DOK2*	*0.0013*
GO:0030282	*Bone mineralization *	*CLEC3B, WNT11, FGFR3, TUFT1*	*0.0014*
GO:0008277	Regulation of G-protein coupled receptor protein signaling pathway	*GNG4, RGS18, RGS9,* *RAMP1*	*0.0022*
GO:2000147	Positive regulation of cell motility	*CCR7, CSF1R, TWIST1*	*0.0037*
GO:0006909	Phagocytosis	*CEBPE, CD93, ELANE, PECAM1*	0.0039
GO:0035335	*Peptidyl-tyrosine dephosphorylation*	*PTPN6, PTPN7, PTPRK,PTPRF, DUSP6*	*0.0042*
GO:0034394	Protein localization to cell surface	*WNT11, ANGPTL1, PTPRK*	0.0051
GO:0043066	*Negative regulation of apoptotic process*	*GLI3, WNT11, ANGPTL1, ANGPTL4, CSF1R, DHRS2, TWIST1, MYC*	*0.0068*
GO:0006468	*Protein phosphorylation*	*FES, MOK, WNT11, CDK14, LMTK3, TRIB3, RPS6KA4*	*0.024*
GO:0060548	*Negative regulation of cell death*	*WNT11, CST3, MYC*	*0.030*
GO:0030097	*Hematopoiesis*	*ANGPTL1, CSF1R, GFI1*	*0.034*
GO:0008285	*Negative regulation of cell proliferation*	*PTPN6, PTPRK, GL13, CSF1R, DHRS2, DLG3CBFA2T3*	*0.048*
GO:0042493	*Response to drug*	*FOS, COL1A1, CST3, HDAC5, MYC*	*0.062*
GO:0001525	*Angiogenesis*	*ANGPTL1, ANGPTL4, PECAM1, RAMP1, MCAM*	*0.096*
GO:0007017	*Microtubule-based process*	*TUBB2A, TUBB3*	*0.10*
	**Molecular Functions **		
*GO:0004725*	*Protein tyrosine phosphatase activity*	*PTPN6, PTPN7, PTPRF, PTPRK, DUSP6*	0.0038
*GO:0005001*	*Transmembrane receptor protein tyrosine phosphatase activity*	*PTPN6, PTPRF, PTPRK*	0.0051
*GO:0030246*	*Carbohydrate binding*	*CLEC3B, CLEC4B, PRG2, LGALS12*	*0.036*
*GO:0004672*	*Protein kinase activity *	*MOK, TRIB3, CDK14, LMTK3, STK33, MAP3K9*	*0.078*
*GO:0008201*	*Heparin binding *	*CLEC3B, ELANE, PTPRF, PRG2*	*0.081*
*GO:0004674*	*protein serine/threonine kinase activity*	*MOK, SBK1, LMTK3, MAP3K9, RPS6KA4, STK33*	*0.091*
*GO:0008013*	*Beta-catenin binding *	GLI3, DACT3, PTPRK	0.095
*GO:0003700*	*Transcription factor binding*	*FOS, PBX2, HDAC5, TWIST1, MYC*	*0.100*
	**Pathways **		
	*Transcriptional misregulation in cancer*	*CEBPE, LMO2, CSF1R, CDK14, MYC, ELANE*	*0.0014*
	*MAPK signaling pathway*	*FOS, PTPN7, MYC, RPS6KA4*	*0.010*
	*PI3K-Akt signaling pathway*	*DDIT4, GNG4, ANGPTL1, COL1A1, CSF1R, FGFR3, MYC*	*0.041*
	*Pathway in cancer *	*FOS, GNG4, GLI3, WNT11, CSF1R, FGFR3, MYC*	*0.069*
	*Hippo signaling pathway*	*WWCI, WNT11, MYC, DLG3*	*0.10*
	*Proteoglycans in cancer*	*WNT11, PTPN6, TWIST1, MYC*	*0.18*
	*Ras signaling *	*GNG4, ANGPTL4, CSF1R, FGFR3*	*0.23*
	*Phagosome*	*TUBB2A, TUBB3*	0.10

(DAVID software analysis, EASE score, p< 0.1)

**Identification of Differentially Expressed Genes by Venn Diagram Configuration**

In MV4-11, out of 9 common differentially expressed genes between TSA, 5-Aza and TSA+5-Aza treatments, 8 genes (*DEF8*,* GUSBP1*,* TUBA1C*,* NDUFC2*,* ARIH2*,* STX12*,* MAP3K6*, and* RAB12*) were commonly up-regulated, while *HEMGN* was commonly down-regulated in all treatments. Between TSA and 5-Aza treatments, *SOCS3* and *HIST1H2BN* were commonly up-regulated, but *PTPRC*,* GIMAP2*,* TPM2*, *CASP4*,* RUNX1-IT1*, and *STAT6* were commonly down-regulated. 16 genes were commonly up-regulated in both 5-Aza and TSA+5-Aza treatments (*FAM200B*,* RBM17*,* C1orf50*,* TMEM120A*, *SETX*,* NAGPA*, *MCUR1*,* BBS4*, *ATG4A*,* SUGP2*, and* RHEB). *5 down-regulated genes in 5-Aza (*FAM133A*, *GPR125*,* GNG11*, *REN*, and* HBD*) shared common down-regulation with TSA+5-Aza treatments. No gene in common was differentially expressed between TSA and TSA+5-Aza treatments. 25, 16 and 38 genes were exclusively expressed in TSA, 5-Aza and TSA+5-Aza, respectively as shown in Figure 3(a) (*p*<0.05).

In Kasumi 1, there were 3 common differentially expressed genes across all treatments; 2 genes (*ANGPTL4 *and* TUBB2A***) **and 1 gene (*ADAM12*) were commonly up-regulated and down-regulated, respectively. Whereas *PTPN6* was either up-regulated in 5-Aza treatment or down-regulated in TSA. *VSTM1* and *KIF1A* were commonly down-regulated in 5-Aza and TSA+5-Aza treatments. There were 36 genes commonly expressed in TSA and TSA+5-Aza treatments with 20 up-regulated and 16 down-regulated genes. 7, 41 and 31 genes were exclusively expressed in TSA, 5-Aza and TSA+5-Aza, respectively as shown in Figure 3(b) (*p*<0.05).

**Figure 3(a):**
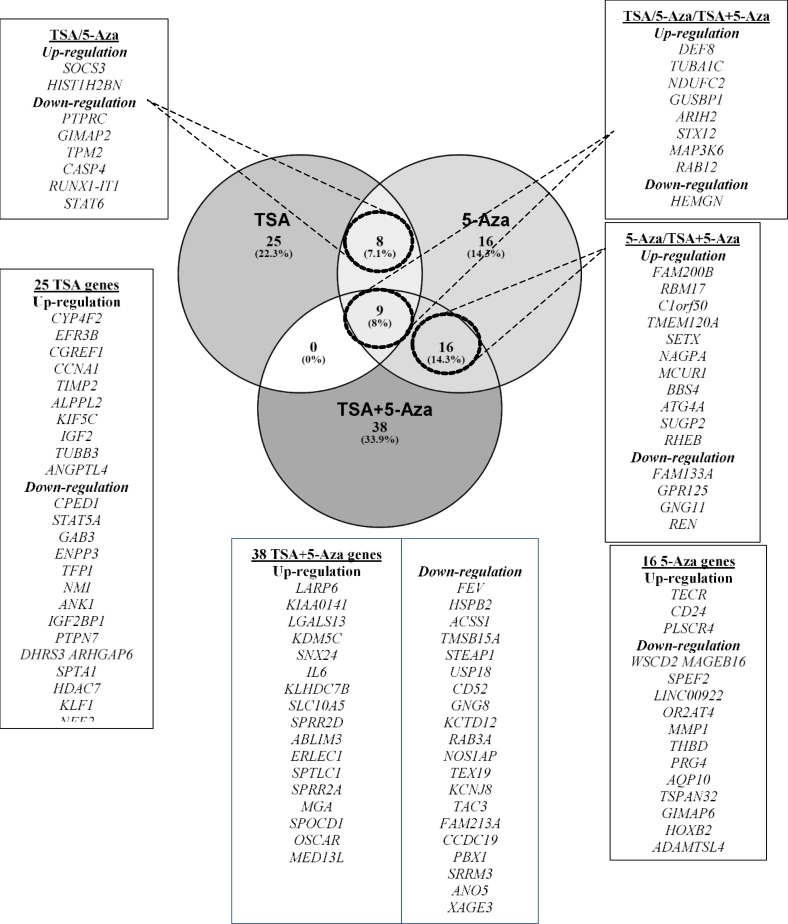
Venn diagram illustrating the genes commonly and exclusively expressed after TSA, 5-Aza and TSA+5-Aza treatments in MV4-11 (adhered to gene selection criteria).

**Figure 3(b):**
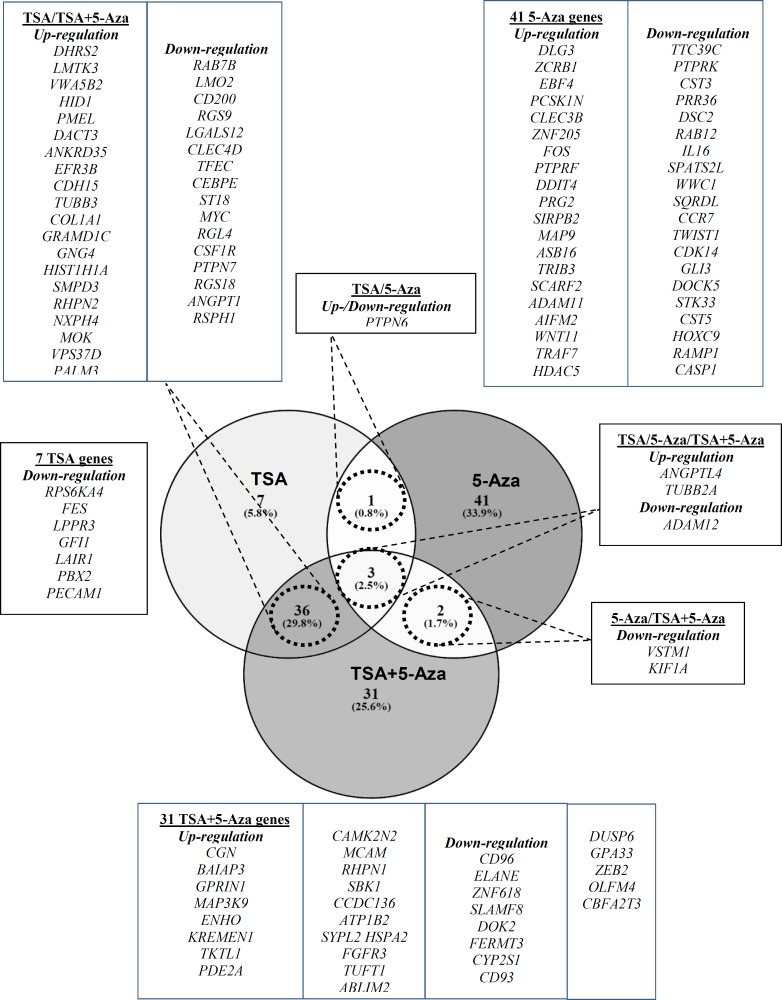
Venn diagram illustrating the genes commonly and exclusively expressed after TSA, 5-Aza and TSA+5-Aza treatments in Kasumi 1(adhered to gene selection criteria).

**Quantitative real-time PCR (qRT-PCR)**

**Figure 4:**
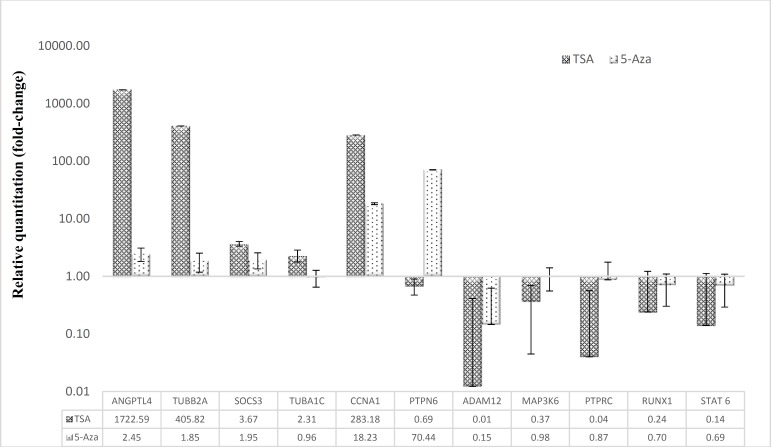
Validation of expression levels of selected genes by qRT- PCR

To verify the expression of genes, commonly up-regulated genes; *SOCS3*, *TUBA1C*,* CCNA1*, and *MAP3K6* in MV4-11; *ANGPTL4 *and *TUBB2A* in Kasumi-1, and commonly down-regulated genes;* STAT6, PTPRC* and *RUNX1* in MV4-11, *ADAM12* and differentially expressed gene, *PTPN6* in Kasumi 1 were selected for validation by qRT-PCR. The results were consistent with that of microarray in both MV4-11 and Kasumi 1 cell lines except for *MAP3K6 *in MV4-11 (Figure 4). 

The qRT-PCR results revealed a significant up- and down regulation of several genes in MV4-11 and Kasumi 1 treated with TSA and 5-Aza compared to non-treated cell lines. *GAPDH *and* B2M* were used as endogenous controls to which the expression was normalized. Shown in the bar graph is the standard error (SE) of duplicated samples.

## Discussion

 It was recognized that epigenetic changes serve as a mediator in cancer progression by the changes of gene expression. Epigenetic alterations are reported to concurrently disrupt the essential signaling pathway predisposed cell to uncontrolled growth, longer survival, and metastasis^   14 ^. Histone modifications and DNA hypermethylation are two known epigenetic mechanisms that largely impact the regulation of gene transcription. Histone modification by acetylation has been found to be significantly deficient in acute leukemia patients, compared with the normal individual^   15 ^. In this study, TSA acts by increasing the acetylation level by inhibiting HDAC activity in human leukemic cell lines. Histone acetylation is known to enhance the expression of specific genes that elicit extensive cellular morphology and metabolic changes, such as growth arrest, differentiation, and apoptosis^   16 ^. 

Aberrant DNA methylation was the most common epigenetic alteration in leukemia in which an increased level of DNA methylation was observed in AML at remission^        17 ^. 5-Aza reverts DNA methylation to induce antineoplastic activity either by global hypomethylation and direct cytotoxicity on abnormal hematopoietic cells in the bone marrow^   18 ^. 5-Aza inhibits DNMT thus to induce re-expression of the silenced genes to halt tumor growth, and to cause modest differentiation in transformed leukemic cell lines and primary AML^   19 ^. The current study found that both TSA and 5-Aza inhibit the growth of MV4-11 and Kasumi 1 cell lines in a dose-dependent manner. The IC_50_ of both treatments at 24 hours were lower in MV4-11, compared to Kasumi 1 which could suggest the inhibitory effect of the drugs were less sensitive in Kasumi 1 harboring t(8;21) than in MV4-11 with *FLT3-*ITD mutation. The variation in the IC_50_ values would also represent different expression signature in response to TSA and 5-Aza treatments. 

It is proposed that the genes which were commonly expressed within TSA, 5-Aza and TSA+5-Aza treatments were epigenetically regulated and involved in the pathogenesis of AML and may serve as candidates for potential biomarkers although they did not share similar GO profile and targeted different signaling pathways. *DEF8, NDUFC2, GUSBP1, ARIH2, STX12 *and* HIST1H2BN *were highly re-expressed (more than 100 folds) in either treatment of MV4-11, have not been previously discussed on their role in cancer except for* HIST1H2BN*. *DEP8* is located at chromosome 16 encodes for an activator of intracellular signal transduction reported to carry single nucleotide polymorphism (SNP) rs4268748 at 16q24 with significantly associated with cell cycle regulator, CDK10 expression^   20 ^. *GUSBP1* which was located at chromosome 5 were involved in transcriptional regulation by putative alternative promoters (PAPs)^        21 ^. *ARIH2* primarily functions in neuronal differentiation was found to be tumor-specific in Glioblastoma multiforme (GBM) correlated with growth suppression in GBM cell lines^   22 ^. Treatment with 5-aza-2′-deoxycytidine resulted in gene re-expression of *HIST1H2BN *in malignant ovarian cancer^   23 ^. Differential down-regulation of *HIST1H2BN *was observed in meningiomas was associated with malignant progression^   24 ^. *RAB12* is a member of RAS oncogene family, function as small GTPase for intracellular protein transport, activated in stimulus-dependent pattern and promote microtubules-dependent of the cell secretary-granule in mast cell^   25 ^ and its up-regulation has been linked with colorectal cancer^   26 ^. 

The most optimal GO in MV4-11 were Cytoskeleton organization involving* TUBA1C*, JAK-STAT cascade involving *SOCS3 *and* STAT6* and the cell cycle involving *CCNA1*, associated with Phagosome, JAK-STAT pathway and Viral carcinogenesis, respectively, *CCNA1* was expressed after TSA treatment with high fold-change (298.44) in MV4-11, but was slightly re-expressed at a low level in 5-Aza and combination treatment (fold-change: 5.67 and 2.81, respectively) (results not shown). *CCNA1,* located at chromosome 13, encodes for activating regulatory subunit which binds to cyclin-dependent kinases 2 (*CDK2)* and cell division cycle 2 (*CDC2)* for the cell cycle machinery to progress into S phase^   27 ^. In normal cells, *CCNA1* was prominently expressed in testes, hematopoietic cells, and brain^   28 ^. *CCNA1* acts as tumor suppressor gene (TSG) which is epigenetically silenced by hypermethylation in cervical cancer^   29 ^, ovarian, renal and lung carcinoma^        30 ^. In AML, *CCNA1* was found to be overexpressed especially in M3 and M2 AML with significant worse overall survival^   31 ^. In addition, upregulation of *CCNA1 *was observed in leukemic cells in response to DNA damaging agents by increasing DNA repair process^   32 ^. *SOCS3*, located at chromosome 17 is the known mediators in the JAK-STAT pathway which is strongly related to AML pathogenesis due to its function in blood lineage differentiation, apoptosis, and proliferation^        33 ^. *SOCS1*, *SOCS2* and *SOCS3* negatively regulate JAK-STAT signaling in AML patients carrying a *FLT3-ITD* mutation^        34 ^. *SOCS3* has been extensively studied for over 20 years for their role in various diseases, especially in cancer. The most widely reported in *SOCS3* was aberrant methylation affecting gene expression and protein function. Hypermethylation of promoter region of *SOCS3* resulted in gene silencing implicated in cancer pathogenesis including hematological malignancies^   35 ^, prostate cancer^   36 ^, pancreatic cancer^   37 ^, endometrial carcinoma^   38 ^, hepatocellular carcinoma^   39 ^ and breast cancer^        40 ^. Other candidate genes convoluted in the JAK-STAT pathway associated with hematological malignancies are *STAT6* and *RUNX1*. *TUBA1C, *located at chromosome 12 is a member of tubulin family of microtubules ubiquitously expressed in the esophagus, bone marrow, appendix, brain, colon, bladder and placenta^   41 ^.* TUBA1C* expression was significantly increased in hepatocellular carcinoma (HCC) on both mRNA and protein level, which predict a poor prognosis^   42 ^, reduced expression in breast cancer associated invasive stage^   43 ^ and their expression was susceptible to colorectal cancer risk ^   44 ^. Cytochrome P450 (*CYP4F2*) was the highest re-expressed gene in TSA treatment with more than 1000 fold-change in MV4-11. *CYP4F2 *is a drug-metabolizing enzyme gene reported to have an epigenetic regulatory role with clinical implication^   45 ^. Inhibition of DNMT and histone deacetylase (HDAC) by 5-Aza and TSA induced the demethylation of *CYP1A1* and *CYP1A2* leading to their up-regulation^   46 ^. 

In Kasumi 1, three common differentially expressed genes in either treatments were *ANGPTL4, TUBB2A*, and *ADAM12* associated with angiogenesis, microtubule-based process, and cell-adhesion, respectively. *ANGPTL4*, located at chromosome 19 encodes a glycosylated, secreted protein containing a fibrinogen-like C-terminal domain, mainly induced by a nuclear receptor protein, peroxisome-proliferator-activated receptor (PPAR)^   47 ^. It is the most studied among *ANGPLT* family, functions primarily in the regulation of lipid metabolism, glucose homeostasis, and insulin sensitivity^   48 ^. *ANGPTL4* has not been previously discussed in the context of AML. However previous studies have reported *ANGPTL4* in various cancer types, including breast cancer, colorectal cancer, prostate cancer, hepatocarcinoma, and renal cell carcinoma, suggesting its important roles in cancer cell growth and progression^   49 ^. In the current study, *ANGPTL4* was mutually up-regulated in TSA treatment in both MV4-11 and Kasumi 1 cell lines, thus has wide potential for gene-specific therapy in AML. *TUBB2A*, located at chromosome 6 is another putative gene in AML with cell-specific expression. It forms a class ll beta-tubulin from six families of tubulins, including, alpha, gamma, delta, epsilon and zeta, and their protein may localize in extracellular exosome, cytoplasm and nucleus, involved in small GTPase activity, GTP binding, nucleotide binding acetylation and methylation^   50 ^. Alpha and beta tubulin sub-families were studied for mutational analysis in human brain tumor and malformations was found in *TUBB2A* affecting the spectrum of "tubulinopathy" phenotypes^51^^, ^^52^. Mutations in *TUBB2A* were also explored in epilepsy^   51 ^, gastric carcinoma and lung cancer^   53 ^ but not hematological malignancies. *ADAM12*, located at chromosome 10 was over-expression in non-Hodgkin’s lymphoma that lead to accelerate of proliferation and cell-adhesion^   54 ^ and was commonly methylated in chronic lymphocytic leukemia^        55 ^. The roles of *ADAM12* in leukemia pathogenesis is still obscure and need further study since the expression of this gene was similarly down-regulated in both treatments. *PTPN6 *(or *SHP1*) located at chromosome 12 was differentially regulated in TSA and 5-Aza treatments (re-expressed only in 5-Aza but not TSA). Our previous study showed a positive correlation of *PTPN6* re-activation due to hypomethylation in MV4-11 that carry a *FLT3*-ITD mutation after the 5-Aza treatment^   56 ^. *PTPN6 *expression has been studied in lymphoma, leukemia and other cancers such as breast cancer, ovarian cancer, prostate cancer, and pancreatic cancer^        57 ^*, *and in hepatocellular carcinoma^   58 ^. *PTPN6* is a downstream mediator in the JAK-STAT pathway, and together with *SOCS3* they potentially serve as molecular indicators for pathway-targeted therapy in AML. Another example of the methylation-related gene is *PRG2*. In the Venn diagram, *PRG2* was exclusively expressed in 5-Aza treatment, but not in TSA treatment. The differentially expressed *PRG2* was reported in three human leukemic cell lines (K562, THP1, and HL-60)^   59 ^. We also previously reported that the expression of *PRG2* was restored after 5-Aza treatment in PKC-412 (Midostaurin) resistant leukemic cell line^   56 ^. *DHRS2* and *LMTK3* were another highly up-regulated genes in TSA treatment in Kasumi 1 with up to 500 fold change. Their up-regulation was due to histone acetylation. 

Finally, despite thousands of genes generated by microarray expression profiling, the highly re-expressed and down-expressed genes perceived in this study were thought to be convoluted with epigenetic regulation of gene transcription in AML. Although only several genes were selected for validation by qRT-PCR, there were many other genes as discussed earlier that may have important roles in cancer pathogenesis. 

## CONCLUSION

In conclusion, we have identified common differently expressed genes that are importants in epigenetic regulation of AML. Our finding also revealed that Phagosome pathway was the most optimal and common in both MV4-11 and Kasumi 1 AML cell lines. Although MV4-11 and Kasumi 1 transduced different optimal signaling pathways in response to drug treatment, it was shown that MV4-11 mainly targeted the genes in the JAK-STAT signaling, while Kasumi 1 targeted the genes in transcriptional misregulation in cancer, PI3K-Akt and MAPK signaling, which are all critical pathways in oncogenesis. These were due to their different molecular characteristics (*FLT3*-ITD vs t(8;21) AML1-ETO). The data presented here may serve as a preliminary finding and are useful for further study to explore epigenetic involvement in the pathogenesis of AML. 
